# Complexion-mediated martensitic phase transformation in Titanium

**DOI:** 10.1038/ncomms14210

**Published:** 2017-02-01

**Authors:** J. Zhang, C. C. Tasan, M. J. Lai, A. -C. Dippel, D. Raabe

**Affiliations:** 1 Max-Planck-Institut für Eisenforschung, Max-Planck-Strasse 1, 40237 Düsseldorf, Germany; 2 State Key Laboratory for Mechanical Behavior of Materials, Xi'an Jiaotong University, Xi'an 710049, China; 3 Department of Materials Science and Engineering, Massachusetts Institute of Technology,77 Massachusetts Avenue, Cambridge, Massachusetts 02139, USA; 4 Deutsches Elektronen-Synchrotron DESY, Notkestrasse 85, D-22607 Hamburg, Germany

## Abstract

The most efficient way to tune microstructures and mechanical properties of metallic alloys lies in designing and using athermal phase transformations. Examples are shape memory alloys and high strength steels, which together stand for 1,500 million tons annual production. In these materials, martensite formation and mechanical twinning are tuned via composition adjustment for realizing complex microstructures and beneficial mechanical properties. Here we report a new phase transformation that has the potential to widen the application window of Ti alloys, the most important structural material in aerospace design, by nanostructuring them via complexion-mediated transformation. This is a reversible martensitic transformation mechanism that leads to a final nanolaminate structure of *α*″ (orthorhombic) martensite bounded with planar complexions of athermal *ω* (*a*–*ω*, hexagonal). Both phases are crystallographically related to the parent *β* (BCC) matrix. As expected from a planar complexion, the *a*–*ω* is stable only at the hetero-interface.

## Abstract (web summary)

One way of tuning mechanical properties of alloys lies in utilizing athermal phase transitions. Here authors report a complexion-mediated martensitic transformation in Ti alloys yielding a nanolaminate structure of martensite bounded by planar complexions, promising new strategies for the design of high strength Ti alloys.

Typical Ti alloys contain primarily phases with hexagonal lattice structure, the so-called *α*-Ti^1^. Alloying can be used to stabilize the high-temperature body-centred cubic titanium phase also at ambient temperatures. These so-called *β*-titanium alloys have become a completely new material class due to their exceptional mechanical properties, namely ultralow elastic stiffness and gum-type deformation behaviour^2^. These are unique advantages, both for biomedical and aerospace applications, which no other alloy class can deliver. The underlying transformation mechanisms occurring in these alloys, which are held responsible for some of their key mechanical properties, are however not understood.

The new transformation phenomenon is observed in *β*-titanium alloys of Ti-23Nb-0.7Ta-2Zr, at.%. This system is a variation of the gum metal^2^, which has drawn significant interest due to its unusual mechanical properties (low elastic stiffness and nearly hardening-free plasticity) and various deformation mechanisms that have been controversially reported as possible underlying reasons for such behaviour (bulk shearing^3^,^4^,^5^,^6^, stress-induced *α*″ martensitic phase transformation^4^,^7^,^8^, dislocation plasticity^9^,^10^, deformation twinning^11^,^12^, *ω*-phase transformation^13^ and strain glass transition^14^). The *β*-phase's instability, when exposed to mechanical loads, evidently plays a critical role in such gum alloys^15^,^16^; therefore, we aim at understanding the phase transformations in this system better. The observed mechanism involves a so far undiscovered coupling of several athermal phase transition steps at the interface, namely, *β*→*α*″ martensitic phase transformation and *β*→*a*–*ω* transition, creating planar complexions at the *α*″/*β* interfaces as well as nano-layered martensitic twinning (Fig. 1). It results in a final nanolaminate composite microstructure throughout the bulk Ti gum metal.

A planar complexion^17^,^18^,^19^,^20^,^21^,^22^,^23^,^24^,^25^,^26^,^27^,^28^ is referred to a metastable interfacial state confined and stabilized in the interfaces of the adjacent phases^20^. It can adopt states that have distinct interfacial structures and/or composition profiles correlated with local minima in free energy^21^ and may undergo reversible structural transitions when the thermodynamic conditions vary^19^,^27^. The complexions known so far mostly appear at grain boundaries and at planar hetero-interfaces, and originate from diffusional transitions^27^. In contrast, the new *a*–*ω* planar complexion observed in this study is induced by a diffusionless martensitic transformation (*β*→*α*″), that is, it forms through a diffusionless process. The confinement and reversibility of this interface nanolaminate, consisting of adjacent orthorhombic *α*″, hexagonal *ω* and twinned *α*″ nanolayers inside the host *β*-matrix realize a planar complexion state. A new transformation-induced planar complexion of *ω*-structure is found. It possesses the athermal character and reversibility through the reversible *β*→*a*–*ω* transition. It forms from the *β*-matrix to accommodate the interfacial strain of *α*″/*β*-phase boundaries during *β*→*α*″ martensitic transition and further mediates the transition by influencing the twinning mode, and results in a final nanolaminate composite microstructure throughout the bulk Ti alloy on cooling.

In what follows, employing various experimental tools (for example, high-resolution transmission electron microscopy (HRTEM), *in-situ* synchrotron X-ray diffraction (SXRD), atom probe tomography (APT) and so on), we provide evidence of the structure, the reversibility and athermal nature of the individual interface features and transformation phenomena involved.

## Results

### Characterization of the phase transformation

**Figure 2:**
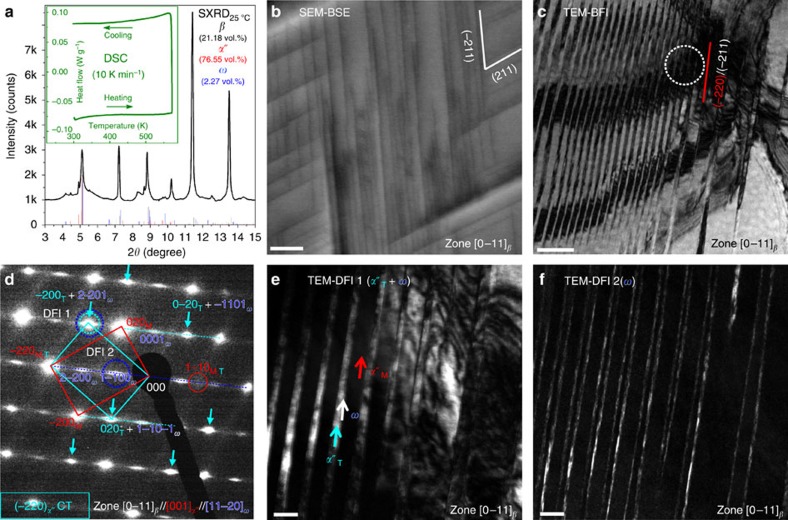
Characterization of the complexion-mediated martensitic phase transformation. (**a**) One-dimensional SXRD pattern, with the DSC inset showing no clear thermal peaks. (**b**) SEM-BSE micrograph (scale bar, 2 μm) from a [0–11]_*β*_ grain shows two sets of {220}_*α*″_ CTs (bulk sample). (**c**) TEM–bright-field image (BFI) shows a set of CT (scale bar, 100 nm). (**d**) Corresponding SADP from the area encircled in **c**. (**e**,**f**) DFIs (TEM–DFI 1 and 2) from the overlapping spot (cyan and blue dotted circles in **d**) and from the *a*–*ω* spot (blue circle in **d**), which indicate that *a*–*ω* planar complexion only locate along the CT boundaries (both scale bars, 20 nm). CT, compound twin.

We start the analysis with the characterization of the phase transformation occurring during quenching (Fig. 2). Figure 2a shows a one-dimensional (1D) SXRD pattern integrated over the two-dimensional (2D) SXRD pattern collected on an area detector from the as-quenched state. It indicates that the high-temperature *β*-phase partially transforms to *α*″ martensite and *a*–*ω* upon quenching. Phase fractions are estimated employing Rietveld refinement of the diffractogram (Fig. 2a) to be 2.27, 76.55 and 21.18 vol.% for *a*–*ω*, *α*″ and retained *β*, respectively (fitting results are presented in Supplementary Fig. 1). The lattice parameters of each structure are also calculated from the SXRD pattern (listed in Supplementary Table 1). Interestingly, the differential scanning calorimetry (DSC) curve (inset of Fig. 2a) shows no thermal peaks upon heating/cooling between 25 and 300 °C, even at the *a*–*ω* and *α*″ reverse transformation finishing temperatures (200 and 245 °C, determined by *in-situ* SXRD results presented later in Fig. 4). The absence of DSC peaks, despite the presence of large volume fractions of *α*″ and *a*–*ω* at room temperature, suggests that both transformation processes take place over a large temperature range, that is, they have a diffuse character.

Figure 2b shows a scanning electronic microscopy–backscatter electrons (SEM-BSE) micrograph of a [0–11]_*β*_ grain (the following lattice correspondences are used in the present paper: *β***→***α*″: [100]_*α*″_–[100]_*β*_, [010]_*α*″_–[011]_*β*_, [001]_*α*″_–[0–11]_*β*_; *β***→***a*–*ω*: [11–20]_*a*–*ω*_–[0–11]_*β*_, [0001]_*a*–*ω*_–[−111]_*β*_). Two sets of edge-on *α*″ twins are observed along {−220}_*α*″_/{−211}_*β*_, indicating that the two edge-on {−211}_*β*_ planes (that is, (−211)_*β*_ and (211)_*β*_) around the [0–11]_*β*_ zone axis can both serve as twinning plane for *α*″ martensite (that is, (−220)_*α*″_ and (220)_*α*″_). In Fig. 2c, TEM–bright-field image shows fine *α*″ twins with (−220)_*α*″_/(−211)_*β*_ twinning plane. For understanding the present twinning mode, the selected area diffraction pattern (SADP) from the [0–11]_*β*_//[001]_*α*″_//[11–20]_*a*–*ω*_ zone axis is presented in Fig. 2d. The {110}_*α*″_ faint spots (shuffle spots), which are more clearly visible in the 2D SXRD patterns shown later, indicate the existence of the *α*″ martensite. Diffuse scatterings with intensity concentration at 1/3{211}_*β*_ positions are the characteristics of the *ω* structure. However, it is unlike the typical diffraction pattern of thermally induced *a*–*ω* (formed during quenching without the assistance of an athermal *β*→*α*″ transformation), where two sets of 1/3{211}_*β*_ reflections are present and of similar intensity^29^,^30^. Only one set is present here and, moreover, half of the 1/3{211}_*β*_ spots have much higher intensity than the others as indicated by cyan arrows (Fig. 2d). To understand the origin of the different intensities of the 1/3{211}_*β*_ spots, two dark-field images (DFIs), referred to as TEM–DFI 1 and DFI 2, were taken by using one brighter and one weaker 1/3{211}_*β*_ spot as shown in Fig. 2e,f, respectively. The TEM–DFI 1 in Fig. 2e shows that one of two martensite laths is bright and the other is dark. The boundaries between them are also bright but in much lower intensity. This indicates that both the bright laths and the boundaries contribute to the brighter 1/3{211}_*β*_ spots. More interestingly, in TEM–DFI 2 (Fig. 2f), only the boundaries are bright. Therefore, the TEM–DFIs reveal that the brighter 1/3{211}_*β*_ spots originate from both *α*″ twin (*α*″_T_) and *a*–*ω*, whereas the ones of lower intensity are formed solely from *a*–*ω* along the twin boundaries. With this, it can be concluded that the SADP in Fig. 2d reveals higher intensity 1/3{211}_*β*_ spots and primary spots correspond to a {−220}_*α*″_ compound twin (CT). It is noteworthy that the {−220}_*α*″_ CT is the only twinning mode found in all TEM observations in this study. This is in contrast with binary Ti-Nb, where the major twinning mode of *α*″ is {111}_*α*″_ type I twinning and no CTs were found^30^,^31^. Moreover, the TEM–DFIs reveal that 1/3{211}_*β*_ spots originate from the preferentially formed *a*–*ω* phase along the pure shear direction {−211}<111>_*β*_ of the *β*→*α*″ transformation^32^. It indicates that the formation of *a*–*ω* is induced by the *β*→*α*″ transformation. As a result, a nanolaminate microstructure is created, composed of sequentially arranged layer-by-layer *α*″ CT bounded with *a*–*ω* films, which resembles a planar complexion state^20^,^27^.

### Atomic configuration of *α*″ CT and *a*–*ω* planar complexion

**Figure 3:**
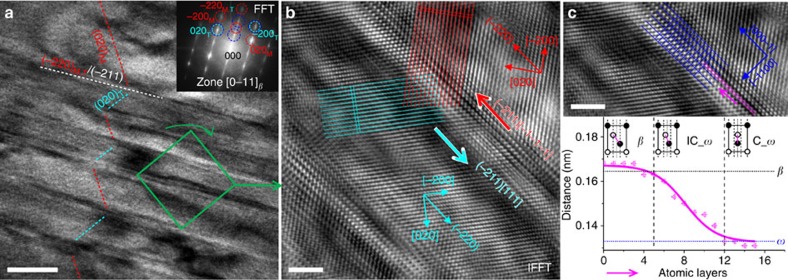
HRTEM images of *α*″ CT and *a*–*ω* planar complexion. (**a**) Low-magnification HRTEM image; the inset shows the corresponding FFT diffraction pattern (scale bar, 10 nm). (**b**) Atomic configuration of *α*″ CT, where red and cyan arrows with white background represent the shear and accommodation directions of *α*″ matrix and its twin during transformation. (**c**) Analysis of atomic configuration within *a*–*ω* planar complexion: *β*→IC_*ω* (incommensurate *ω*)→C_*ω* (commensurate *ω*), where the inset is the plot of atom distance between the middle atoms along the purple lines. Scale bars, 2 nm (**b**,**c**).

Figure 3 shows HRTEM images from the [0–11]_*β*_ zone axis, providing the atomic configuration of the *α*″ CT and *a*–*ω* planar complexion. Figure 3a shows a HRTEM where several successive nanolayers of *α*″ CT bounded by *a*–*ω* planar complexions were captured. The corresponding fast Fourier transform (FFT) pattern is shown in Fig. 3a (inset), which is almost identical to the SADP in Fig. 2d. An inverse FFT image of higher magnification (from the green zone in Fig. 3a) is shown in Fig. 3b,c, focusing on the internal structure of *α*″ CT and *a*–*ω*, respectively. Figure 3b shows the atomic configuration of *α*″ CT, where red/cyan lines along the (020)_M,T_ planes reveal the deviation of the (020)_M,T_ lattice planes across the *a*–*ω* layer, which also indicates the *α*″ strain accommodation direction by *a*–*ω* as denoted by thicker red/cyan arrows (with white background). The lattice spacings in the centre of *α*″_M_ and *α*″_T_ are *d*_(0–20)M,T_=0.24 nm and *d*_(200)M,T_=0.16 nm in agreement with the values obtained from SXRD (Supplementary Table 1). Furthermore, analysis of one *a*–*ω* zone has been performed, as shown in Fig. 3c. As plotted in the inset, the distance between two middle atoms of *a*–*ω* was measured and plotted with respect to the atomic layers along the purple lines. The following structures can be identified: *β*→IC_*ω* (incommensurate *ω*)→C_*ω* (commensurate *ω*)^33^,^34^,^35^. This sequence provides the first clear perception revealing the individual steps contributing to the *β*→*a*–*ω* transformation mechanism. Consequently, the C_*ω* and IC_*ω* zones can be outlined in the *a*–*ω* planar complexion regions as shown in Supplementary Fig. 2. On the boundary of the C_*ω* zones, 1/2<111>{211} dislocations are identified. Moreover, one segment of a twin boundary without *a*–*ω* complexion is found between the two complexion regions outlined, where the interfacial stress falls below a critical value. The histogram of the *a*–*ω* planar complexion-width distribution is shown in Supplementary Fig. 3. It has been calculated based on TEM–DFI 2 (Fig. 2f) for better statistics.

This observation also provides another evidence that the *a*–*ω* planar complexion is induced by a shear stress exerted by the *α*″ transformation, as this shear stress along {−211}<111>_*β*_ (the pure shear of *β*→*α*″ transformation) has a perfect lattice correspondence for promoting the *β*→*a*–*ω* transformation, which will be explained further below. Furthermore, *a*–*ω* plays a role in accommodating *α*″ martensitic twins as manifested by a pair of remaining planar strain feature on both sides of the *a*–*ω* layers (Fig. 3b). Hence, a new complexion/second-phase accommodation mechanism is identified in martensitically transforming materials: the interfacial stress of *β*→*α*″ is accommodated by triggering the formation of planar complexion through *β*→*a*–*ω* transformation.

### Reversibility of the *α*″ CT and *a*–*ω* planar complexion

**Figure 4:**
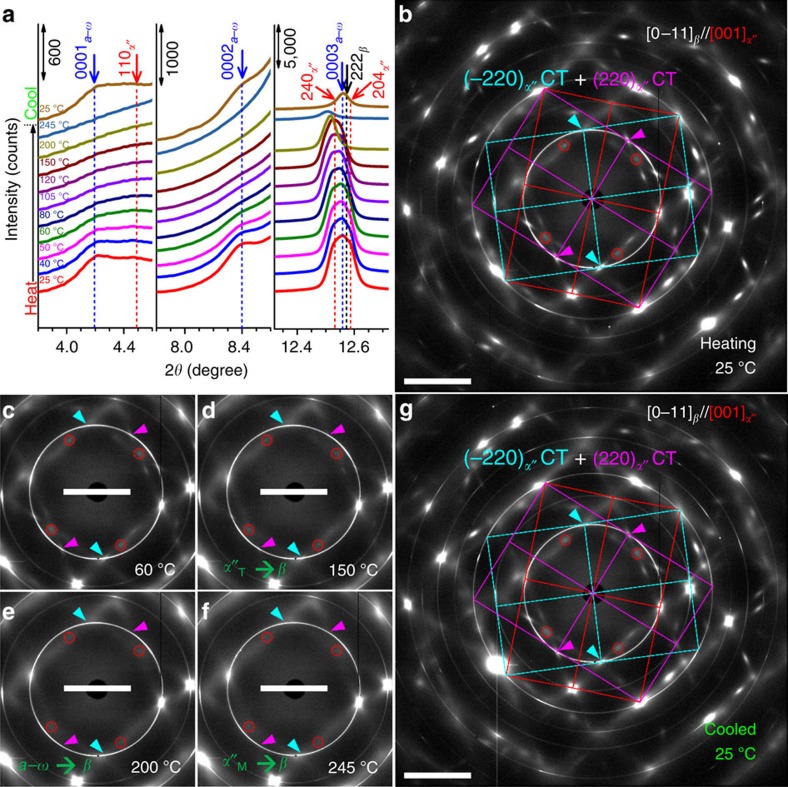
Results of *in-situ* SXRD heating/cooling experiments. (**a**) Line profiles integrated from 2D SXRD patterns. (**b**–**g**) Representative 2D SXRD patterns at 25, 60, 150, 200, 245 °C (during heating) and 25 °C (after cooling), respectively. Scale bar, 5°.

The results of the *in-situ* heating/cooling SXRD experiments shown in Fig. 4 provide another direct evidence for the co-existence and the reversibility of *α*″ CT and *a*–*ω* complexion. Figure 4a shows three segments of the 1D SXRD patterns integrated from the 2D SXRD patterns. Some representative 2D SXRD patterns are shown in Fig. 4b–g. In Fig. 4a, gradual disappearance of the *ω* and *α*″ reflections within 2*θ* ranges of 3.8° to 4.6° and 7.8° to 8.6° during heating, and their restoration after cooling to 25 °C prove the reversibility of *β*→*a*–*ω* and *β*→*α*″ transformations during heating/cooling. Another broad peak within the 2*θ* range of 12.3° to 12.7° consists of (240)_*α*″_(0003)_*ω*_, (222)_*β*_ and (204)_*α*″_ reflections. During heating, the overlapping peaks become narrower at 200 °C, indicating full reversion of *a*–*ω* to *β* around 200 °C, as one *a*–*ω* peak is located at higher 2*θ*. This is also revealed more clearly in the analysis of 2D SXRD patterns, presented in the following section. When the temperature is further increased, the peak becomes even smaller at 245 °C, which is the reverse transformation finishing temperature of *α*″→*β*. Hence, at 245 °C, a single *β*-phase is achieved, which gains even more solid proof through the 2D SXRD pattern captured at 245 °C (Fig. 4e).

Next, the 2D SXRD patterns in Fig. 4b–g are further analysed, to provide more insights on the transformation kinetics and overall sequence of *α*″_T_→*β*, *a*–*ω*→*β* and *α*″_M_→*β* transitions. Based on the grain identification performed on the 2D SXRD pattern at 245 °C (single *β*-phase), as presented in the Supplementary Fig. 4, diffraction intensities in the current *in-situ* SXRD pattern originated mainly from three grains: two of them are [0–11]_*β*_ and the other one is [012]_*β*_. In the following, we focus on the diffraction pattern of one [0–11]_*β*_ grain with near on-pole condition, with its diffraction pattern outlined in green in Supplementary Fig. 4.

In Fig. 4b, a 2D SXRD pattern obtained at 25 °C (before heating) is shown and the primary diffraction spots are linked by red lines to indicate that the grain is mainly in *α*″ martensite state. Additional to the primary spots, extra spots at 1/2{211}_*β*_ and 1/3{211}_*β*_ locations are also found. Based on the SADP analysis shown in Fig. 2d, two sets of {−220}_*α*″_ CT diffraction spots from the [001]_*α*″_//[0–11]_*β*_ zone axis are identified as marked in cyan and magenta, which is consistent with the SEM-BSE image in Fig. 2b. The (020)_*α*″_ twin spots of each CT are indicated by cyan and magenta arrows (pointing to the <111>_*β*_ shear direction of *β*→*α*″ transformation), respectively; in contrast, {1–10}_*α*″_ spots caused by {0–11}<011>_*β*_ shuffling associated with the *α*″ transformation are encircled (red). One noticeable difference between the diffraction patterns of TEM (Fig. 2d) and SXRD (Fig. 4b) is the absence of (1–100)_*ω*_ and (2–200)_*ω*_ spots in the SXRD pattern. These signals are actually ‘forbidden' diffraction spots in the hexagonal lattice structure. Their appearance in TEM SADP (Fig. 2d) is due to the double diffraction effect^36^. In the 2D SXRD pattern, these spots disappear for the following two reasons. (1) The SXRD beamline used for this study has a much larger wavelength (*λ*=0.020727, nm) as compared with TEM operated at 200 kV (*λ*=0.00251, nm) and hence a much smaller radius of the Ewald sphere, which leads to lower double diffraction intensity values. (2) The thickness of the samples used in SXRD is ∼1 mm. In contrast, the TEM thin foils used in this work are below 200 nm in thickness. Therefore, in the SXRD experiments, double diffraction effects drop to near-zero intensity before penetrating the entire sample, different than in corresponding TEM diffraction experiments. For clarity, the key diagrams to this complex diffraction pattern are provided in Supplementary Fig. 5, which consists of two overlapping diffraction patterns from two possible edge-on *α*″ CT: (−220)_*α*″_ CT and (−2−20)_*α*″_ CT. During heating, the intensity of the extra spots due to *α*″ CT and *a*–*ω* decreases gradually (Fig. 4c), as can be seen more clearly in Supplementary Movie 1. In Fig. 4d, the disappearance of twin spots (that is, the intensity of arrow-pointed spots becomes the same as that of the (0001)_*ω*_ *a*–*ω* spots at the smallest 2*θ* angle) at 150 °C indicates that all *α*″_T_ transforms reversely into *β* first around 150 °C. The 2D SXRD pattern taken at 200 °C (Fig. 4e) shows the disappearance of *ω* spots, indicating that the *a*–*ω* fully reverses back to *β* at around 200 °C. When the sample is further heated up, the 2D SXRD pattern at 245 °C (Fig. 4f) reveals that the (1–10)_*α*″_ shuffle spot vanishes, indicating that *α*″ fully transforms back to *β*. After cooling to 25 °C, the 2D SXRD pattern in Fig. 4g shows that the original diffraction pattern (Fig. 4b) is nearly fully restored. This *in-situ* heating/cooling SXRD analysis proves the complete reversibility of *α*″ CT and its associated *a*–*ω* complexion.

**Figure 5:**
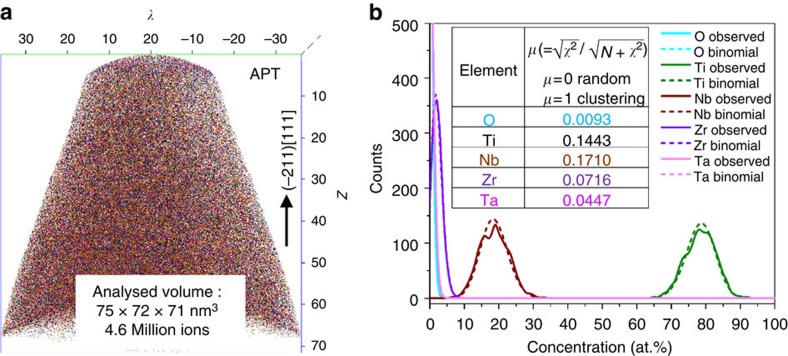
APT analysis. (**a**) APT elemental map displays the atomic-scale local composition. (**b**) Frequency distribution curves for Ti, Nb, Ta, Zr and O atoms obtained from the APT data shown in **a**; binomial distributions for average solute contents are also shown for comparison, which stands for the ideal distribution in a homogenized alloy.

The results mentioned above also suggest that the {−220}_*α*″_ CT-induced *β*→*a*–*ω* transformation is martensitic, hence without composition change. To confirm this, APT analyses were carried out on tips that were focused ion beam (FIB) milled from a {111}_*β*_ grain. The cluster analysis reveals that all alloying elements are distributed homogenously, indicating an athermal transformation mechanism (Fig. 5).

## Discussion

**Figure 6:**
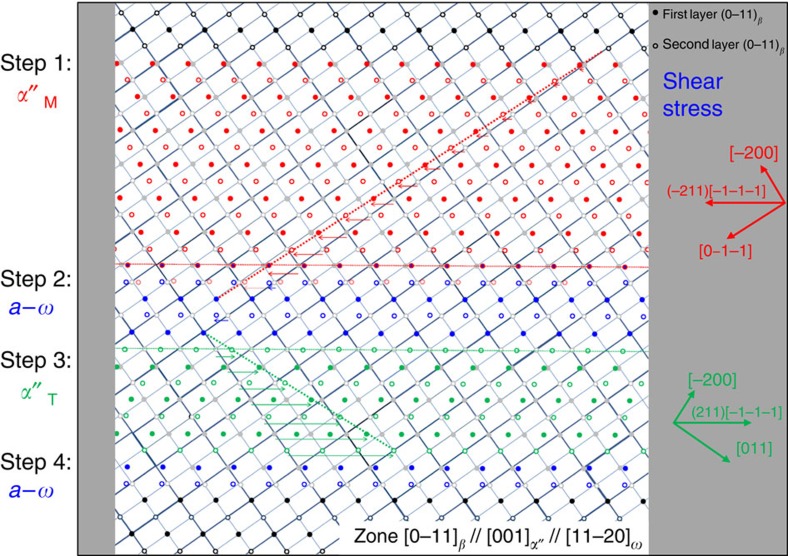
Schematic representation of atomic movements associated with the complexion-mediated transformation upon cooling. Open and solid circles represent the first and second layers of atoms viewed from [0–11]_*β*_ orientation. Colour in the figure: black to *β*-phase; blue to *a*–*ω*; red to *α*″-matrix; green to *α*″-twin. For better clarity, only *β*-indexes are used here. The transformation starts from the top and propagates to the bottom during cooling as divided into four steps: Step 1 (formation of *α*″-matrix), Step 2 (formation of first *a*–*ω* layer), Step 3 (formation of *α*″-twin) and Step 4 (formation of second *a*–*ω* layer).

To elucidate the underlying mechanisms of the *a*–*ω* planar complexion mediated transformation, a schematic representation of the transformation process on cooling is presented in Fig. 6. When the *β*→*α*″ transformation starts (Step 1), the (−211)[−1−1−1]_*β*_ shear stress at the lower interface of the *α*″_M_ and *β* is built up as indicated by the red arrows. Simultaneously, confining back-stresses from the surrounding untransformed *β* matrix are built up, acting against the shear direction of *α*″_M_, the extent of which is indicated by the height of blue energy bands on both sides. These two stress components act in opposite directions, creating a compressive stress on the (−211)_*β*_ plane along the effective axis [111]_*β*_ of the *a*–*ω* formation^35^,^37^. Growth of *α*″ stops and *a*–*ω* forms when the stress reaches a critical value required for inducing *β*→*a*–*ω* (as illustrated in Supplementary Fig. 6a,b). This marks Step 2, the occurrence of which indicates that *a*–*ω* is thermodynamically not stable but can be stabilized under the assistance of stress. Formation of the *a*–*ω* planar complexion accommodates the strain (evident in Fig. 3b) and hence decreases the interfacial stress. When the local stress drops to values below the critical value, the growth of the *a*–*ω* layer stops, as the *a*–*ω* region is still thermodynamically unfavourable. This is also the reason why the occurrence of the *β*→*a*–*ω* transition is strictly confined to the interface region (forming a planar complexion of a size around 2 nm) and not viable outside of it. As the *a*–*ω* transformation produces shear along the (−211)[−1−1−1]_*β*_ component, *α*″_T_ of the {−220}_*α*″_/{−211}_*β*_ compound twinning character is formed (Step 3) due to the requirement of interface energy minimization. The second *a*–*ω* layer forms (Step 4) also because of a planar compressive stress accumulated by the *β*→*α*″_T_ transition. However, the formation of the *α*″_T_ involves very large shear stresses (due to 35% twinning shear strain) along (−211)[−1−1−1]_*β*_ and hence the critical stress of the transformation *β*→*a*–*ω* is reached ‘faster' (in fewer atomic layers) than that caused by *α*″_M_ in Step 1. Moreover, the formation of the second *a*–*ω* layer stops the further growth of the *α*″_T_ layer. As a results, the width of the *α*″_T_ layer should be much thinner than that of the *α*″_M_ layer, which is consistent with the presented TEM observations (Fig. 2e). The accommodation potential of both *a*–*ω* layers are the same and therefore the two *a*–*ω* layers should have similar thickness (evident in Fig. 2f). These four individual structural steps are considered as one transformation event, mediated by a planar complexion state at the interface. With further cooling, the final nanolaminate microstructures can be formed after repeating the four-step transformation event, as illustrated in Supplementary Fig. 6c,d. Two more SEM-BSE images in Supplementary Fig. 7 provide a larger field of view of such nanolaminate microstructures from both [0–11]_*β*_ and [111]_*β*_ orientations. Such a nanoscale successive transformation process, when progressing over a wider temperature range, shows no abrupt thermal signal as is evident from the DSC measurement (inset in Fig. 2a). This phenomenon is similar to the disappearance of the DSC peak observed during the strain glass transition^38^. Based on the above discussion, it is quite clear that the formation of *a*–*ω* planar complexions is to accommodate *β* → *α*″ interfacial strain and also mediates the *β*→*α*″ transition to possess {−220}_*α*″_ compound twinning character. It results in a final nanolaminate composite microstructure throughout the bulk Ti gum metal.

In conclusion, a new reversible complexion-mediated martensitic phase transformation phenomenon has been discovered in titanium, which is mediated by a planar complexion state. It involves the joint formation of the {−220}_*α*″_/{−211}_*β*_ α″ martensitic CT and the accommodating *a*–*ω* planar complexion. TEM analysis shows that the two transformation steps are coupled, constituting a complexion interfacial state: the *a*–*ω* transformation is induced by a shear stress initiated by pure shear {−211}<111>_*β*_ during *β*→*α*″, which has perfect lattice correspondence for inducing the *a*–*ω* complexion. *In-situ* heating/cooling SXRD and APT prove that both *α*″ and *a*–*ω* transformations are reversible and martensitic. This coupled and complexion-mediated transformation mechanism enables novel nanostructuring and hence strengthening opportunities for Ti alloys.

## Methods

### Alloy synthesis and heat treatment

A reversible complexion-mediated martensitic phase transformation is observed in the oxygen-free gum metal (Ti-23Nb-0.7Ta-2Zr, at.%) upon quenching. The actual composition of the alloy was determined by chemical analysis as Ti-23Nb-0.67Ta-1.96Zr-0.26O (at.%). The chemical composition was tested by inductively coupled plasma optical emission spectrometry and infrared absorption spectroscopy measurements. The melt of the ingot was produced under argon atmosphere in an arc-melting furnace from pure elements, cast into a copper mould, homogenized at 1,200 °C for 4 h and then furnace cooled. Smaller samples of the alloy were further annealed in vacuum quartz tubes for 1 h at 1,000 °C and subsequently water quenched as the tube was broken simultaneously, to avoid iso-*ω* formation^39^.

### Synchrotron measurements

SXRD measurements were performed on the bulk samples with a thickness of ∼1 mm, at beam-line P02.1 at PETRA III (DESY Hamburg, Germany) with the wavelength *λ*=0.020727, nm^40^. Beam size used was 500 × 500 μm^2^. The 2D SXRD patterns were collected on an area detector (PerkinElmer XRD1621). Azimuthal integration was performed using the software *FIT2D* and the obtained 1D SXRD patterns were then analysed by Rietveld refinement using MAUD^41^. The 1D SXRD pattern in Fig. 2a indicates the co-existence of *α*″ and *a*–*ω* phases, and a small volume of remaining *β*-phase. The *a*–*ω* reflections are quite diffuse/broad and low in intensity. The patterns are rather complex, owing to the overlap with the *α*″ and *β* peaks, which will fail fitting or lead to wrong fitting results. Hence, the *a*–*ω* reflections were not included during the first-step Rietveld refinement (shown in Supplementary Fig. 1a), through which a successful fitting (*R*_w_=9.0%) was reached and the volume fractions of *α*″ and remaining *β*-phase were determined to be 82 and 18 vol.%, respectively. By using first step refinement as the starting point, second step Rietveld refinement with *a*–*ω* reflections added, converged well (*R*_w_=7.84%), as presented in Supplementary Fig. 1b. From this analysis we suggest the room temperature phase fractions for *a*–*ω*, *α*″ and *β* as 2.27, 76.55 and 21.18 vol.%, respectively. *In-situ* heating/cooling SXRD experiments were carried out under the same condition with a home-made heating stage as illustrated in Supplementary Fig. 8.

### Physical and microstructural characterization

The DSC measurement was performed in a Mettler Toledo DSC1 at heating/cooling rate of 10 °C min^−1^. The sample was first heated up from room temperature (25 °C) to 300 °C and subsequently cooled down to 25 °C. TEM samples were electro-polished (Struers Tenupol 5) at 6 °C using an electrolyte of A3. TEM observations were performed in a JEOL JEM-2200 FS at an acceleration voltage of 200 kV, through which bright-field images, DFIs, SADPs and HRTEM images were recorded by a Gatan SDD Camera. FFT and inverse FFT analysis were applied to the HRTEM images. SEM-BSE and electron backscatter diffraction (EBSD) were carried out in a Zeiss Crossbeam XB 1540 FIB-SEM instrument (Carl Zeiss SMT AG, Germany). EBSD was used to determine the grain orientation where SEM-BSE micrographs were taken. To maintain consistency with the notation used for the TEM results, [0–11] and [111] are used to denote the plane normals of <110> and <111> grains determined by EBSD. Elemental distribution at atomic scale was studied using local electrode APT (LEAP 3000X HR, Cameca Inc.). APT tips were prepared from a [111]_*β*_ grain (as shown in Supplementary Fig. 7b), following the standard procedure^42^ in a FEI Helios Nanolab 600i dual-beam FIB.

### Data availability

The data that support the findings of this study are available from the corresponding authors on request.

## Additional information

**How to cite this article:** Zhang, J. *et al*. Complexion-mediated martensitic phase transformation in Titanium. *Nat. Commun.* **8,** 14210 doi: 10.1038/ncomms14210 (2017).

**Publisher's note**: Springer Nature remains neutral with regard to jurisdictional claims in published maps and institutional affiliations.

## Supplementary Material

Supplementary Information Supplementary Tables and Supplementary Figures

Supplementary Movie 1 A movie of 2D SXRD patterns collected during in-situ heating/cooling SXRD experiments. The patterns provide direct evidence for the co-existence of a"_CT_ and a-ω at 25 ^o^C as well as their reversibility upon heating to 245 ^o^C and subsequent cooling to 25 ^o^C.

## Figures and Tables

**Figure 1:**
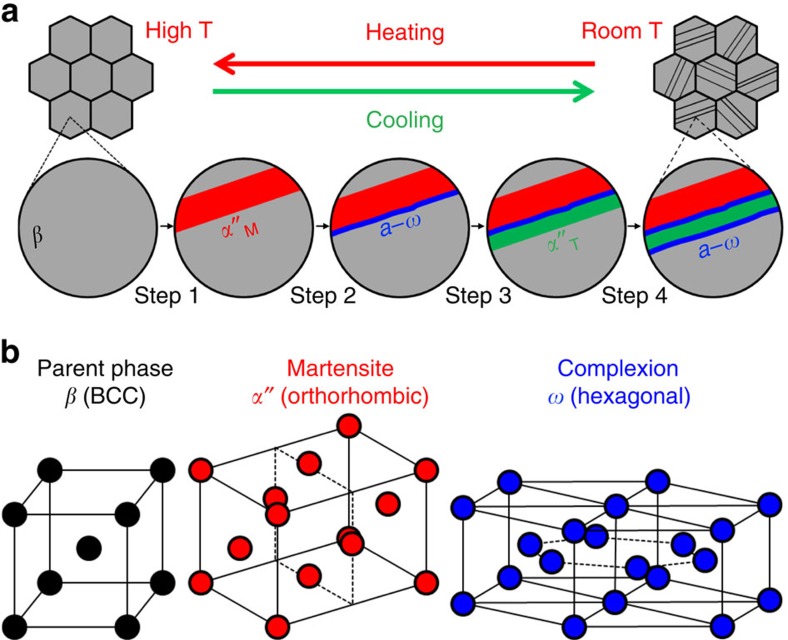
Schematic illustration of the phase transformations and crystal structures of the phases involved. (**a**) Schematic illustration for complexion-mediated martensitic phase transformation in a *β*-Ti alloy matrix (from left to right): Step 1, formation of *α*″-matrix; Step 2, formation of first *a*–*ω* planar complexion; Step 3, formation of *α*″-twin; and Step 4, formation of second *a*–*ω* planar complexion. (**b**) Crystal structures of *β* parent phase, *α*″ martensite and *ω* complexion.
