# Supplementary material for: Complexion-mediated martensitic phase transformation in Titanium
Source: Nat Commun. 2017 Feb 1;8:14210. doi: 10.1038/ncomms14210 (PMC5296643; doi:10.1038/ncomms14210)
Supplement: Supplementary Information — Supplementary Tables and Supplementary Figures [file ncomms14210-s1.pdf]

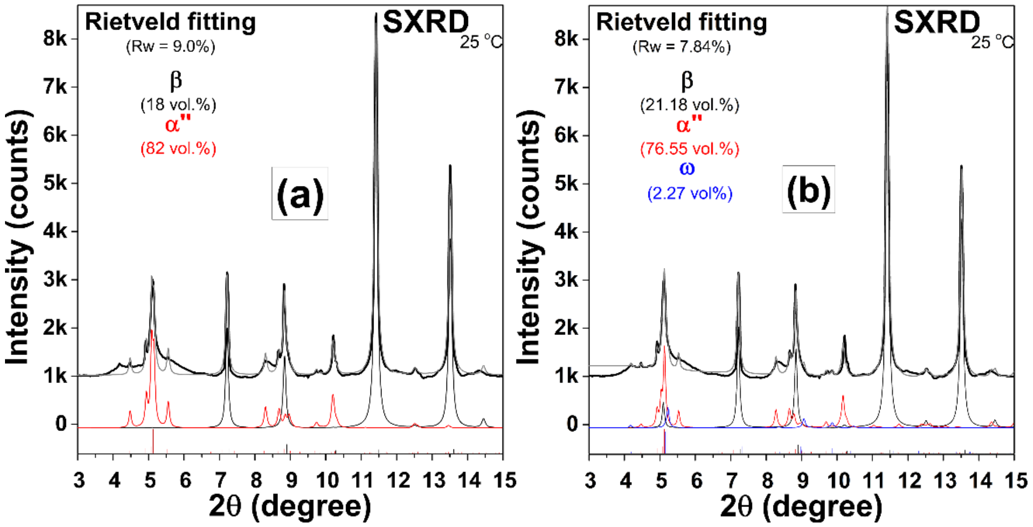

Supplementary Figure 1: Rietveld fittings of 1D SXRD pattern in article Figure 2a. (a) 1st step fitting results with β + α" phases; (b) 2nd step fitting results with β + α" + ω phases using the parameters obtained by using 1st step as a starting point.

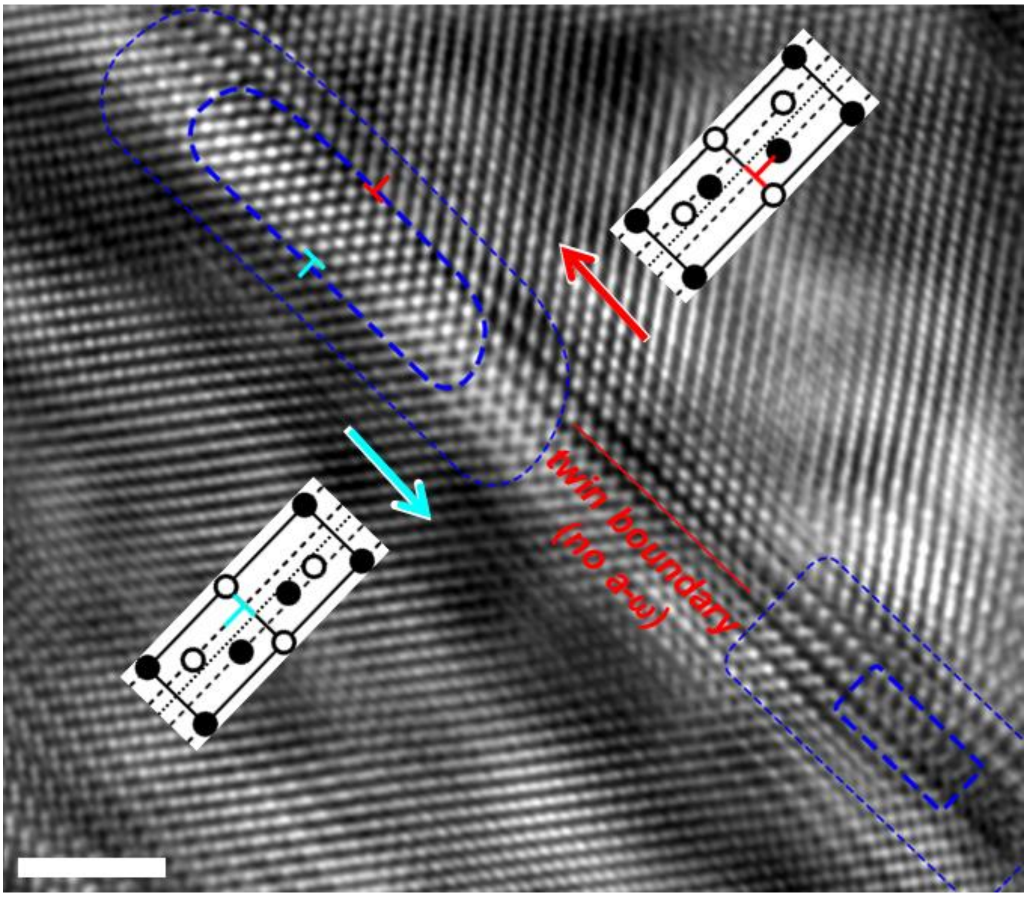

Supplementary Figure 2: Microstructure of two abutting a-ω planar complexion regions. Two a-ω planar complexion regions are outlined with thick (C_ω zone) and thin (IC_ω zone) blue dashed lines in the HRTEM image shown in article Figure 3b. On the boundary of C_ω zones, 1/2<111>{211} dislocations are identified (insets show the schematic illustrations of the dislocations formation during the complexion-mediated martensitic phase transformation). In addition, one twin boundary with no a-ω complexion is found and denoted with red solid line. Scale bar is 2 nm.

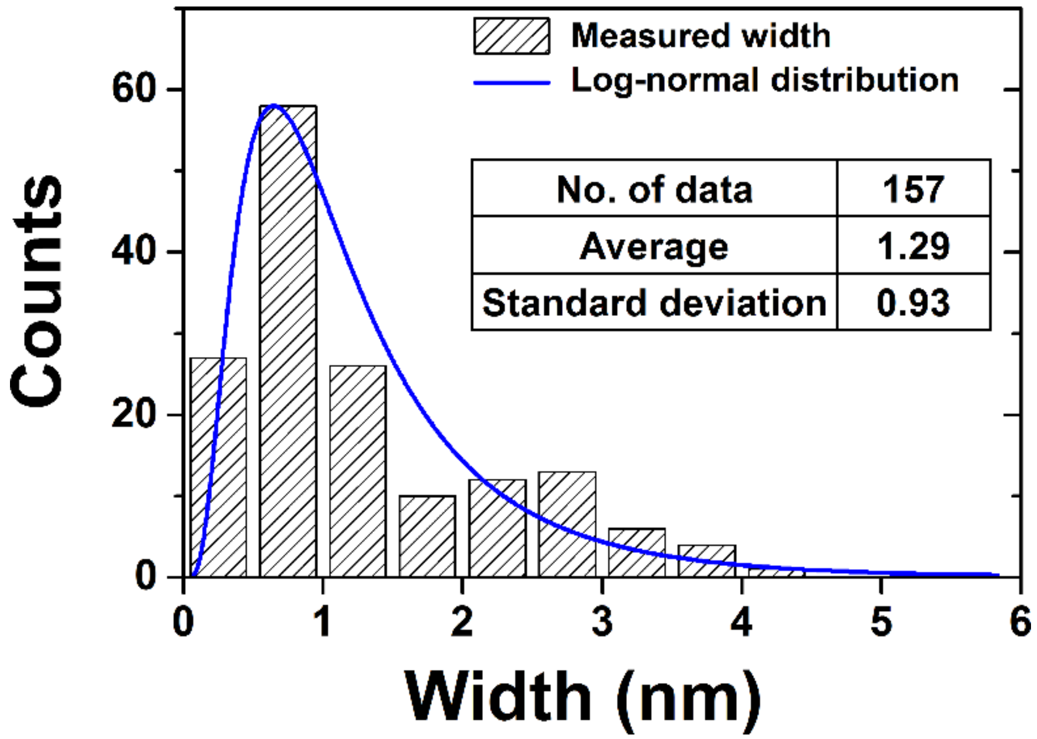

Supplementary Figure 3: Width of a-ω planar complexion. Distribution histogram of the width of a-ω planar complexions (measured from TEM DFI 2 in article Figure 2f), together with a regression curve optimized using the log-normal distribution. The average width of a-ω and standard deviation are calculated to be 1.23 and 0.93 nm, respectively.

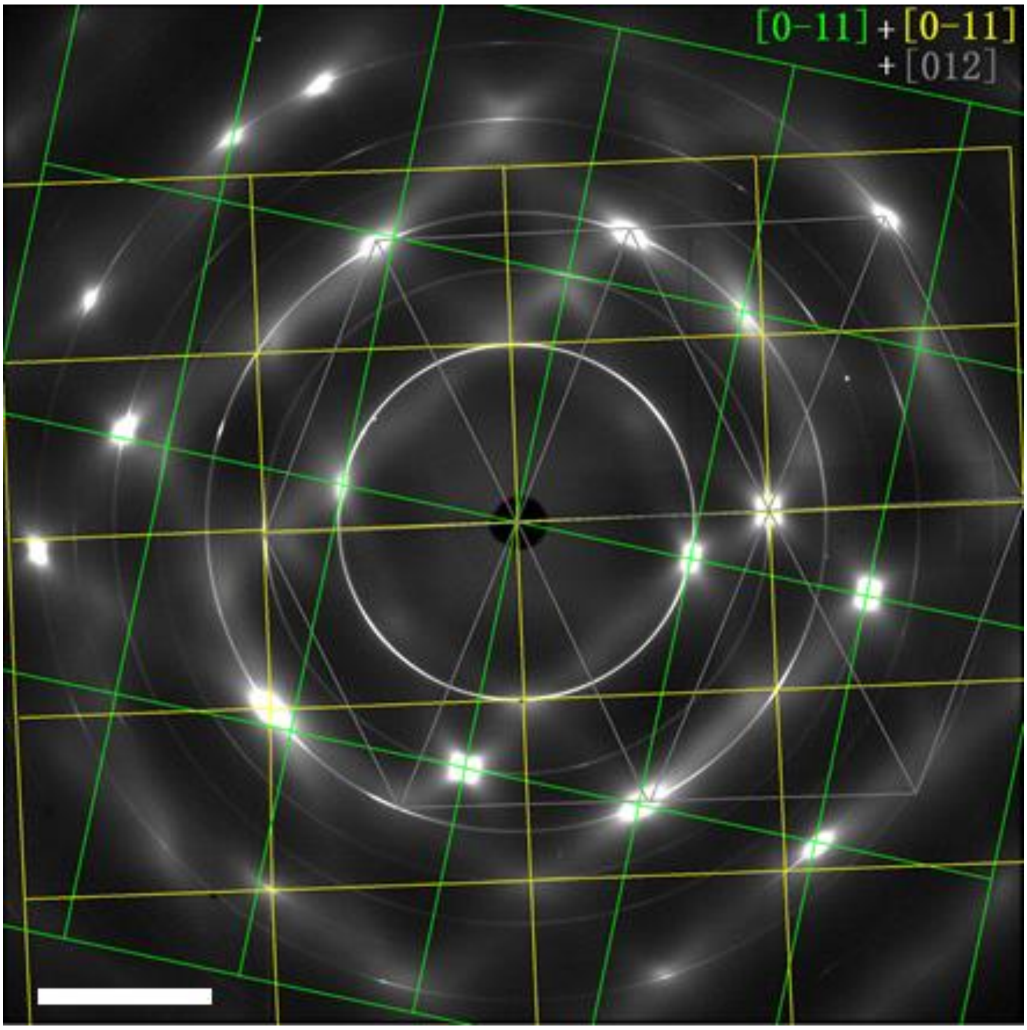

Supplementary Fig. S4: Grain identification for in-situ SXRD pattern analysis. 2D SXRD pattern at T_{max} (245 ^{o}C) of fully reversed β phase state, where the major grains contributing to the diffraction pattern are identified to be two $[0\text{-}11]_{\beta}$ grains (green and yellow lines) and one $[012]_{\beta}$ (grey lines). Scale bar is 5 degrees.

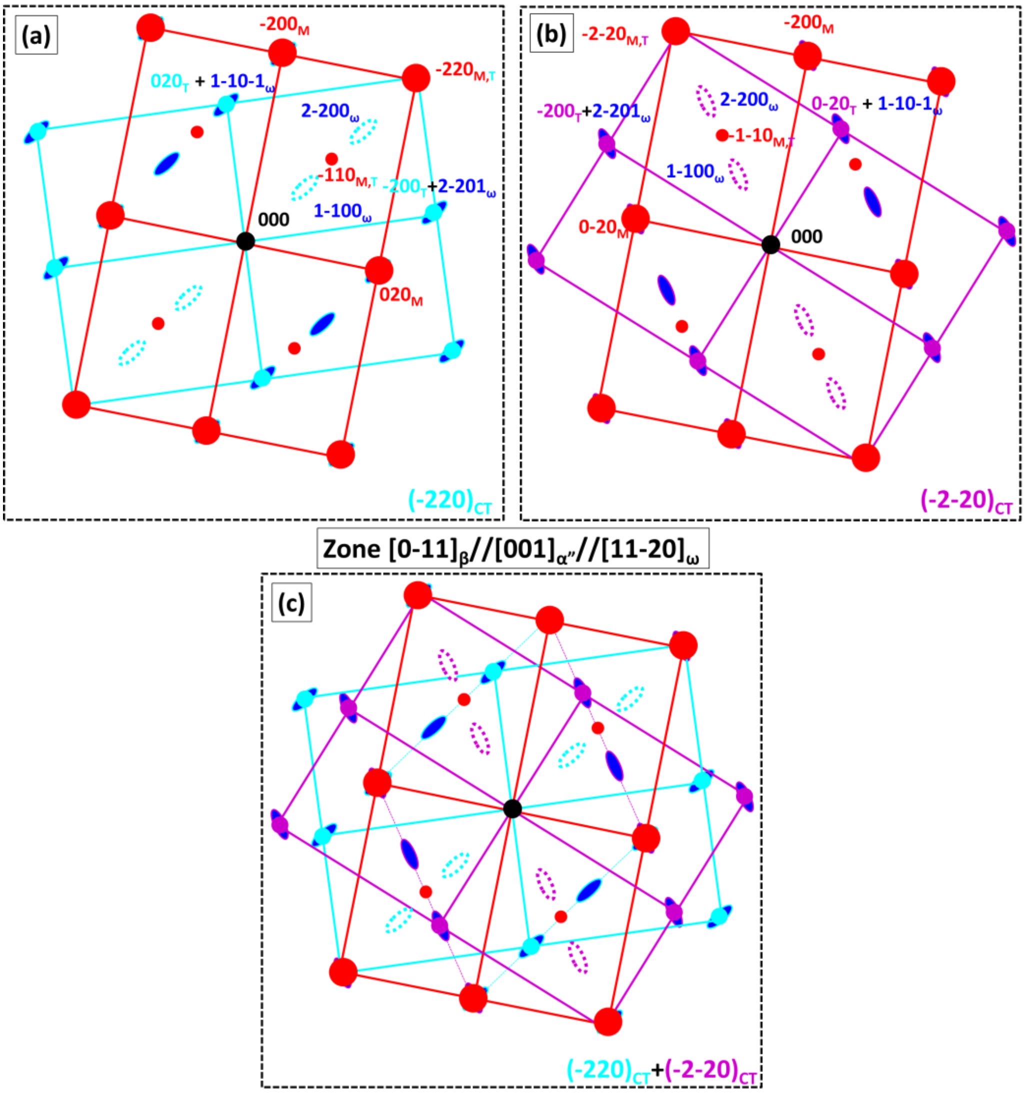

Supplementary Figure 5: Key diagrams to overlapped diffraction patterns from two possible edge-on $\{220\}_{\alpha''}$ CT in $[0\text{-}11]_{\beta}$ grain as in article Figure 4b. (a) Diffraction pattern from $(\text{-}220)_{\alpha''}$ CT. (b) Diffraction pattern from $(\text{-}2\text{-}20)_{\alpha''}$ CT. (c) Overlapped diffraction patterns from $(\text{-}220)_{\alpha''}$ CT and $(\text{-}2\text{-}20)_{\alpha''}$ CT. Color red is designated to α''_{M}, while cyan and magenta denote α''_{T} in $(\text{-}220)_{\alpha''}$ CT and $(\text{-}2\text{-}20)_{\alpha''}$ CT. Elliptic spots are ω spots, where boundary colors are associated to the CT inducing them. Elliptic spots (dashed) are the ω spots extinct in 2D SXRD pattern but present in SADP (article Figure 2d) and FFT (inset of article Figure 3a) patterns in TEM/HRTEM.

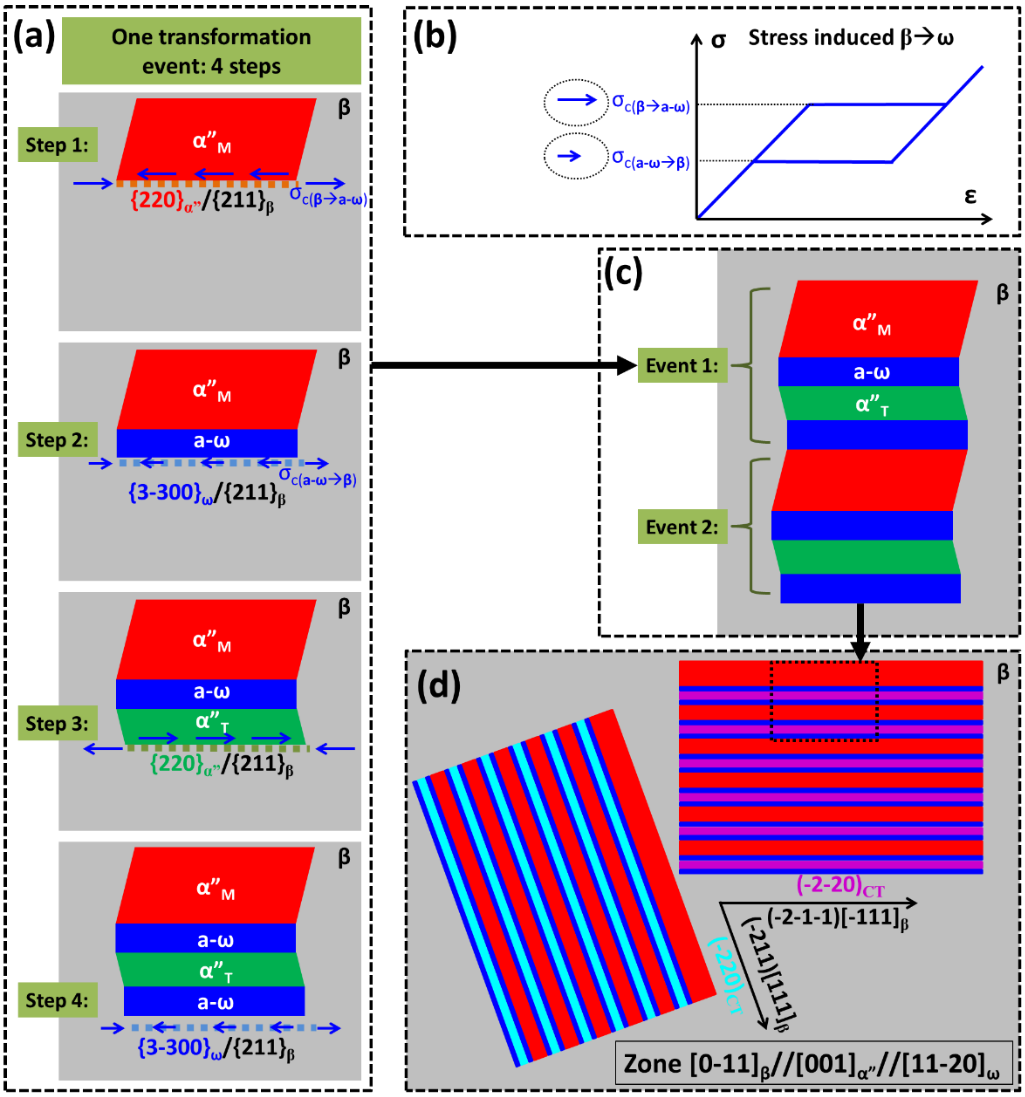

Supplementary Figure 6: Schematic illustration for the formation of nanolaminate through the complexion-mediated martensitic phase transformation. (a) One event of complexion-mediated martensitic phase transformation includes 4 steps as illustrated in article Figure 6. (b) Schematic illustration of stress-strain curve for the stress-induced β↔a-ω transformation, where the thermodynamically unfavorable a-ω planar complexion is induced by shear stress of β→a'' transformation, and also kept stable by the interfacial stress (> $\sigma_{c(a\text{-}\omega\rightarrow\beta)}$) along the $\{220\}_{\alpha''}/\{3\text{-}300\}_{\omega}$ interfaces. (c) Transformation proceeds by repeating 4 steps in (a). (d) Two blocks of nano-composite formed in a $[0\text{-}11]_{\beta}$ grain as shown in Supplementary Figure 7a.

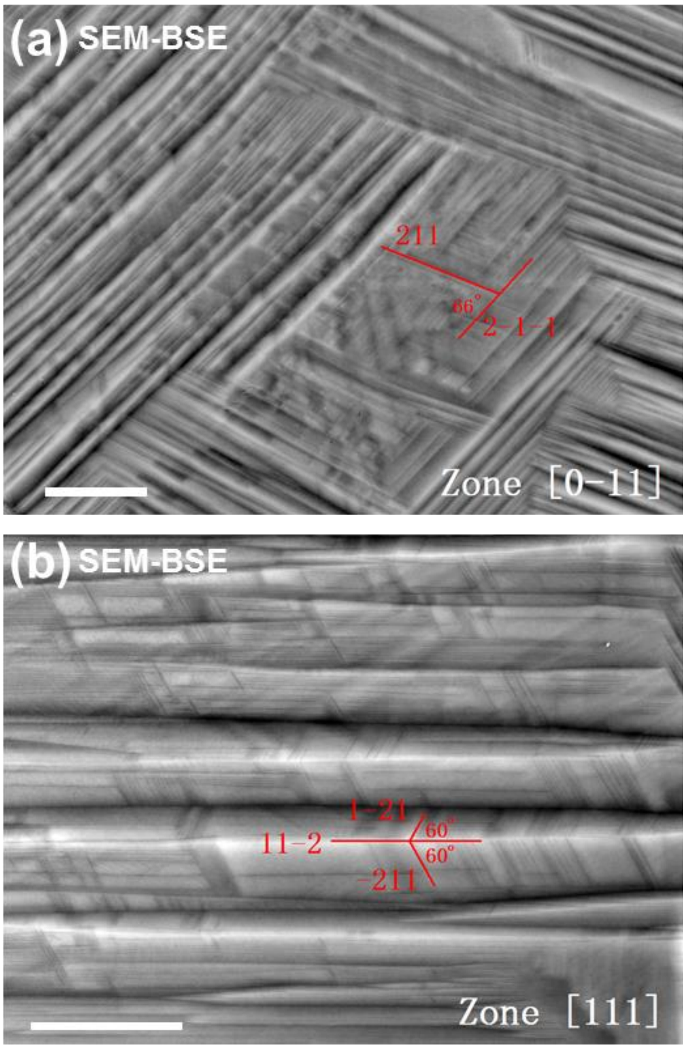

Supplementary Figure 7: SEM-BSE micrographs. α''_{CT} morphology in the bulk sample (electro-polished) from grains of orientation (a) $[0\text{-}11]_\beta$ and (b) $[111]_\beta$, which is determined by EBSD method. The edged-on $\{211\}_\beta$ plane traces are outlined with red lines, the angles between which are also identified. Scale bars are both 10 μm.

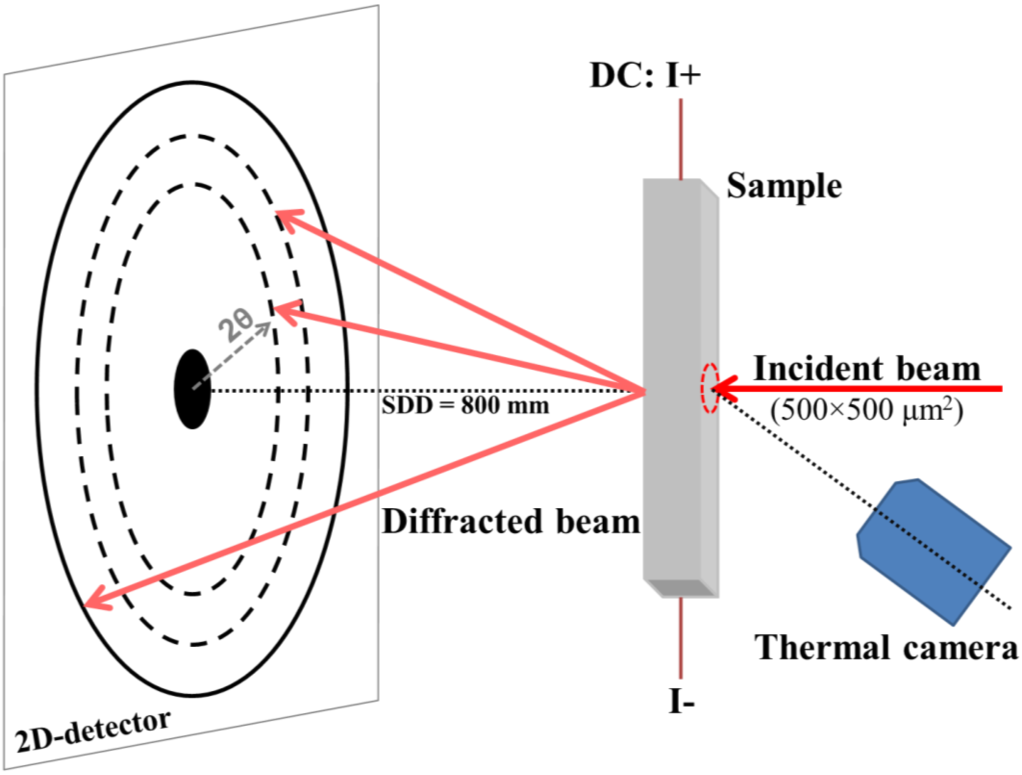

Supplementary Figure 8: Schematic illustration of the in-situ SXRD setup. The sample is heated by controlled direct current (DC) during in-situ heating/cooling SXRD.

Supplementary Table 1: Lattice spacing calculated from 1D SXRD pattern in article Figure 2a. Lattice spacing of a-ω, α” and β phases at 25 °C and transformation strains(%) of a-ω, α” with respect to β. (+) and (-) in strains indicate the shrinkage or expansion of *d*-spacing as compared to the parent β phase.

**Basic planes**	$(100)_{\beta}$	$(100)_{\alpha''}$ / $(100)_{\beta}$	$(010)_{\alpha''}$ / $2*(011)_{\beta}$	$(001)_{\alpha''}$ / $2*(0\text{-}11)_{\beta}$	$(10\text{-}10)_{a\text{-}\omega}$ /$2*(\text{-}101)_{\beta}$	$(0001)_{a\text{-}\omega}$ /$3*(222)_{\beta}$
**Lattice constant (nm)**	0.327	0.320	0.483	0.468	0.458	0.284
**Transformation strain (%)**	**—**	**-2.23**	**+4.40**	**+1.26**	**-1.031**	**+0.183**
**Planes in zone** **$[0\text{-}11]_{\beta}$ / $[001]_{\alpha''}$ / $[11\text{-}20]_{a\text{-}\omega}$**	$(211)_{\beta}$	$(222)_{\beta}$	$(220)_{\alpha''}$ / $(211)_{\beta}$	$(240)_{\alpha''}$ / $(222)_{\beta}$	$(1\text{-}100)_{a\text{-}\omega}$ / $3*(211)_{\beta}$	$(0001)_{a\text{-}\omega}$ / $3*(222)_{\beta}$
**d-spacing (nm)**	0.1347	0.0945	0.1334	0.0964	0.3966	0.284
**Transformation strain (%)**	**—**	**—**	**-0.965**	**+2**	**-0.867**	**+0.183**
